# Place and city: toward a geography of engagement

**DOI:** 10.1016/j.heliyon.2019.e02261

**Published:** 2019-08-16

**Authors:** Albert Acedo, Tiago Oliveira, Mijail Naranjo-Zolotov, Marco Painho

**Affiliations:** Nova Information Management School (NOVA IMS), Universidade Nova de Lisboa, Campus de Campolide, 1070-312 Lisbon, Portugal

**Keywords:** Sense of place, Social capital, Civic engagement, Spatial dimension, Geographies of engagement, Geography

## Abstract

The relationship between sense of place, social capital and civic engagement has been studied in different disciplines. However, their association has been less examined, and their spatial relationship has been analyzed even less. This study contributes to a better understanding of the relationship between these three concepts (i.e., sense of place, social capital and civic engagement). Furthermore, we analyze the crucial role that the spatial relationship between them plays. Using spatial data collected through a web map-based application, we adopt structural equation modeling (SEM) techniques to assess the repercussion that sense of place has on social capital and how the latter affects civic engagement. We find that sense of place is significant and positively correlated with social capital, while the latter also significantly explains civic engagement at the individual level. Furthermore, we observe a better statistical performance in almost all cases when a spatial relationship between the three constructs exists. Our research leverages SEM techniques, Geographic Information Science (GISc) methods, and participatory methodology to show the spatial connection between sense of place and social capital to explain civic engagement. Deriving and quantifying such meaning allows us to highlight the importance of their spatial dimension in city processes such as participation.

## 1 Introduction

The importance of encouraging people to act as participative citizens in issues of public concern is essential for a functioning democracy, particularly when researchers are observing that civic engagement (CE) is diminishing in developed countries (Aricat and Ling, 2016). In turn, the relationship that individuals have toward a certain geographical area (i.e., sense of place (SoP)) or their significant social relationships (i.e., social capital (SC)) embedded within an area can play a crucial role on the engagement of a citizen (Perkins et al., 1996). Researchers have revised the connection between individuals' place attachment and many forms of CE, such as civic activity (Lewicka, 2005), community participation and planning (Manzo and Perkins, 2006) or pro-environmental behavior (Buta et al., 2014). All these studies register the importance of relationships between citizens and their meaningful places, in which they can have significant relationships, to citizens' engagement. However, the association between participation, place, and space has received little attention (Haywood, 2014). Hence, the study of individuals' spatialities (i.e., individual or collectives practices related to their geographical location that reflects their spatial actions and interactions (Lussault, 2007)) regarding SoP and SC in the city context can offer an alternative to better understand and foster participatory processes (i.e., CE). Our approach has its roots in the understanding of cities as place networks (Massey, 1994; Roche, 2016; Acedo et al., 2018) and how we can comprehend a relational space based on networks of actions and actors (e.g., humans, objects) (Murdoch, 1998; Latour, 2005; Duff, 2011). Based on that, the main objective of this study is the research of the (spatial) relationship among SoP, SC, and CE to assess the spatial importance of the first two (i.e., SoP and SC) in the socio-spatial practices of CE (e.g., participatory processes). Our study aims to exalt the spatial dimension (i.e., in this study, the geographical definition on a map of the area that covers the feelings, thoughts and acts toward an object represented through geographic primitives) of individuals' spatialities regarding SoP and SC as an important aspect to better understand CE in the urban context.

This study performs a theoretical literature review to assess the relationship between SoP, SC and CE and their dimensions from a non-spatial perspective. Based on that, we attempt a revision of the same concepts from a spatial point-of-view. In this research, a spatial perspective means to study (1) the spatial imprint of a concept defined by its location and (2) the relative location versus other concepts (i.e., proximity, density). We merge a web map-based approach with traditional questionnaires based on softGIS methodology (Kahila and Kyttä, 2009; Kyttä and Kahila, 2011) to gather the spatial dimension of SoP, SC and CE. We analyze the answers using partial least squares structural equation modeling (PLS-SEM) techniques (Hair et al., 2014) to illustrate their quantitative relationship and assess the potential of considering the spatial dimension of the social concepts (i.e., SoP and SC) to better understand CE in the city context. Our methodology is eminently based on citizens' spatialities associated with SoP, SC, and CE; i.e., the entire methodology revolves around a geographic perspective with a practical focus on studying the social-spatial practices of CE such as participatory processes in local or community affairs in the city context. Therefore, those new spatial contexts can operate shared geographies of engagement that can underpin collaboration, cooperation and interaction between citizens engaged with these specific geographic areas in, for instance, local affairs, social issues or planning decision-making processes.

This research materializes the first step towards these new *“geographies of engagement”* (1) performing a theoretical literature review between SoP, SC and CE and their dimensions, and (2) studying and assessing the influence of SoP on SC and the latter on CE with special focus on when their spatial relationship in a proposed model occurs. This article starts with a review of the SoP, SC, and CE conceptualizations and dimensions. From there, the suitability of understanding those concepts from a spatial point-of-view is reasoned with the declaration of some hypotheses. The article then presents the methods and the results of an experiment conducted in Lisbon (Portugal) to clarify the importance of the spatial dimensions of SoP, SC, and CE to explain their relationship. This explanation is followed by a discussion of the results, the remaining gaps, the limitations, and finally the conclusions of this research.

## 2 Theoretical background and hypotheses

A city can be understood under a relational nature between actions and actors (e.g. humans, objects) (see actor-network theory (Latour, 2005; Law, 2008). Murdoch (1998) specified the characteristics of that city-space, arguing a folded and striated geography in which all action is relational and reflects both the diversity of materials used in construction and the relations between elements. Drawing on the same line of reasoning, Duff (2011) mentioned three needed resources (i.e., social, affective, and material) to enable and define places. The relationship between the three aspects forms networks and flows that configure the city environment. The same author describes the social resource as social capital, the affective resources mean feeling states and action-potential, and the material resource covers the physical aspect of place as well as services and information. Recently, Acedo et al. (2018) also put in value the understanding of a city by platial urban dynamics, arguing the potentiality to conceptualize SoP and SC as inhibitors of place notion based on Agnew, 2002, Agnew, 2011. Those mentioned conceptualizations can apply to any city; the challenge resides on how to operationalize those arrangements in the city context to better understand the urban synergies.

SoP refers to the feelings, beliefs and behaviors that humans associate with a place (Jorgensen and Stedman, 2001). The same authors explicitly argue for positivistic research in the SoP notion and propose three dimensions (place attachment, place identity and place dependence). Place attachment is usually defined as an emotional bond that connects people to places (Altman and Low, 1992; Manzo, 2005; Lewicka, 2013), while place identity refers to the relation between a place and one's personal identity (Proshansky et al., 1983; Trentelman, 2009). Finally, place dependence is the potential of a place to meet the necessities of an individual or group with respect to other places (Jorgensen and Stedman, 2001).

SC analyzes the value of social relationships and networks to societies and individuals (Holt, 2008; Chan, 2018), and it can be analyzed by four dimensions: sense of community, collective efficacy or empowerment, neighboring and citizen participation (Perkins and Long, 2002; Perkins et al., 2002). Sense of community is the feeling of membership to a group (Perkins and Long, 2002), while collective efficacy/empowerment is the belief and thought of the potentiality of acting together. Neighboring encloses the informal actions and behaviors of citizens to a group or society (Acedo et al., 2017b) that essentially occurs in localities (Mahmoudi Farahani, 2016), and citizen participation describes the change from passive to active involvement in the local activities and decisions (Adler and Goggin, 2005) and electronic participation (Naranjo Zolotov et al., 2018).

CE explains associations or ways in which citizens have a common purpose of preserving and promoting public goods (Son and Lin, 2008), to improve conditions for others (Cegarra-Navarro et al., 2014), community (Putnam, 2000) or collective benefit (Moro, 2010). Many times CE is conceptualized as a process rather than an event (UNDP Evaluation Office, 2002), as a measurement of the right of citizens to have a say in the decisions that affect their lives (Sheedy et al., 2008, p. 4).

### 2.1 Relating sense of place, social capital, and civic engagement

**Figure 1:**
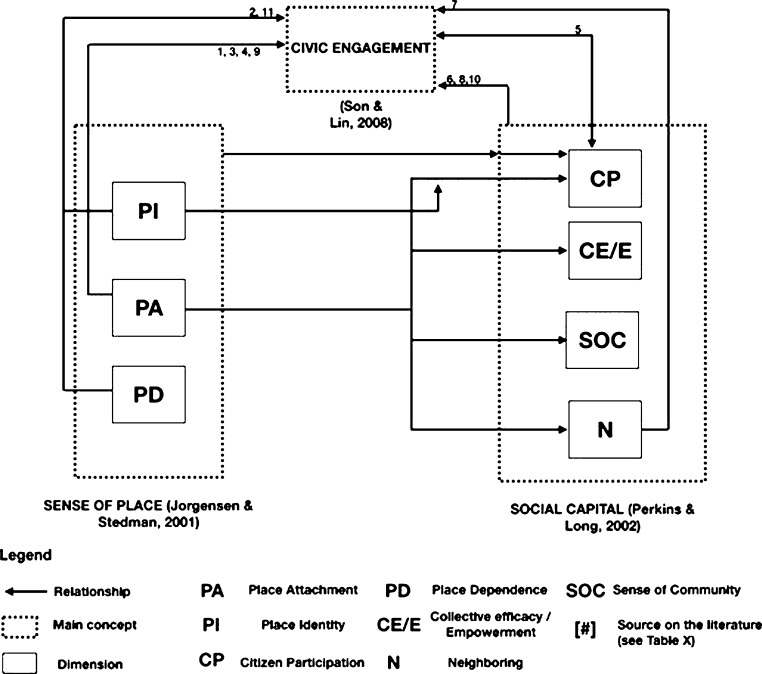
Schema showing the relationships between sense of place and social capital dimensions toward civic engagement. Figure adapted from Acedo et al. (2017b), numbers in the arrows are references listed in Table 1.

A commitment to place motivates SC (Jorgensen, 2010) and neighborhood ties (Lewicka, 2005). Processes of collective action (dimension of SC) perform better when there are emotional ties to places (Manzo and Perkins, 2006). Along in the same line of thought, emotional and behavioral attachment is related to a sense of community (Pretty et al., 2003). Some studies systematically demonstrate the existence of a relationship between SoP and SC (Mesch and Manor, 1998; Jorgensen, 2010; Raymond et al., 2010). For instance, Acedo et al. (2017b) performed a systematic literature review with more than 20 references showing the strong relationships between SoP and SC and their dimensions (based on attitude theory (Rosenberg, 1960; Ajzen and Fishbein, 1975; Fishbein and Ajzen, 1975)). Fig. 1 depicts the connections found between the dimensions of SC and SOP towards CE after performing a theoretical literature review (*see* Table 1).

**Table 1:** Relationship between numbers in Fig. 1 and authors.

Number in Fig. 1	Citation	Author
1	*“Such attachment (attachment to place) motivated interviewees to participate in campaigns against developments that they perceived would threaten these place-based values.”*	(Lin and Lockwood, 2014, p. 80)
2	*“It was therefore predicted that people who protested would have higher levels of place attachment; a prediction confirmed by the significant correlations between protesting and both place identity and place dependence in this study.”*	(Anton and Hons, 2016, p. 151)
3	*“From this we can conclude that while people with strong place attachment […] it is those who also have positive attitudes about the value and importance of pro-testing, who perceive civic action as the norm amongst their friends and family, and who perceive that they have control over their actions that may be more likely to actively oppose place change.”*	(Anton and Hons, 2016, p. 20)
4	*“Specifically, people who were more attached to a place were more likely to express behavioral intentions to engage in place-based planning actions.”*	(Kil et al., 2014, p. 486)
5	*“Although, people participation is affected by civic engagement, but people participation also plays a crucial role in promoting civic engagement, […]”*	(Mohammadi et al., 2011, p. 212)
6	*“individual social capital was the consistent and significant predictor of both expressive and instrumental civic actions.”*	(Son and Lin, 2008, p. 341)
7	*“As the model reported here shows, it is neighborhood ties and not place attachment that predicted civic involvement.”*	(Lewicka, 2005, p. 392)
8	*“civic virtue is most powerful when embedded in a dense network or reciprocal social relations”*	(Putnam, 2000)
9	*“Both community attachment and park related place attachment played a role in predicting citizens' levels of pro-environmental civic engagement beliefs.”*	(Buta et al., 2014, p. 1)
10	*“the connections among individuals such that, over time, a social network is created in which people come to expect mutual support and trust. This leads to: (a) potential increases in each individual's physical health and social–emotional well-being, as well as (b) potential increases in civic engagement and employment in the community of which they are a part, both contributing to a healthier and more effectively functioning society.”*	(Hunter, 2016, p. 200)
11	*“According to the structural model, the influence of place meanings on participatory planning intentions was significant. Specifically, people who were more attached to a place were more likely to express behavioral intentions to engage in place-based planning actions.”*	(Kil et al., 2014, p. 486)

The analysis of Fig. 1 shows the relationships between the central concepts and their dimensions of this research and depicts literature-based evidence that SoP and SC are strongly related to CE. Overall, the PA dimension of SoP is the dimension most related to CE, while when referring to main concepts, SC is the most associated with CE. Therefore, based on the literature reviewed, from a non-spatial perspective, both concepts (SoP and SC) and their dimensions show a plausible connection with CE.

CE can encompass place-based activities (Adler and Goggin, 2005) and involves more direct forms of citizens' participation (Zlatareva, 2008) that contribute to the greater good (Chan, 2018). Chen (2016) distinguishes different forms of CE such as civic, electoral or political activities. In the same vein, Son and Lin (2008) understand CE as a conceptual framework that contains a multitude of elements and measurements. For instance, membership in voluntary organizations, religious participation or membership in civic associations. Both CE and SC incorporate mutual obligation and responsibility for action (Putnam, 2000). In turn, a precondition for CE is the existence of SC (Zlatareva, 2008), since highly attached people are more willing to work collectively to reach a desired goal (Brown et al., 2002). Indeed, recent studies asserted that social capital is positively related to civic engagement (Chan, 2018) and partially impacts e-participation (Naranjo-Zolotov et al., 2019). Interestingly, Haywood (2014) positioned sense of place scholarship as a crucial resource to the better understanding of public participation in scientific research. In turn, Lewicka (2005) proves that it is neighborhood ties (SC dimension) and not place attachment (SoP dimension) that predicts civic involvement. Later, the same author 2011 underlines the inconsistent pattern of relationships between affective bonds toward places and place-focused actions such as participation or planning. Therefore, based on the citations shown in Fig. 1 and the literature of this section, a positive SoP could increase the SC of an individual, and the latter, in turn, could also increase the intention to participate (i.e., CE). Table 2 shows the two research hypotheses fruit of this section.

**Table 2:** Research hypotheses regarding the relationship between sense of place, social capital and civic engagement.

Hypotheses number	Hypotheses
*H*_1_	Citizens' sense of place (SoP) has a positive effect on social capital (SC).
*H*_2_	Citizens' social capital (SC) has a positive effect on their civic engagement (CE).

### 2.2 Relating (spatially) sense of place, social capital, and civic engagement

A recurrent issue studied in the literature is the integration of GISc capabilities in the humanities scholarship (see Bodenhamer et al. (2010)). This synergy is allowing new concepts such as hybrid geographies that are forging creative connections within geographies (e.g., physical and human perspectives) (Sui and DeLyser, 2012). Indeed, this merge highlights the epistemological and social/political meanings inherent in maps and mapping (DeLyser and Sui, 2014) that reinforce the better understanding of how mapping emerges between geographers and social scientists (Kitchin et al., 2013). Conversely, non-representational theorists (e.g., Dewsbury (2003); Thrift (2008)) advocate to not represent the study target as the primary step to extract knowledge (Cadman, 2009) and focus the attention on what cannot be represented (Pile, 2010). In much the same way, Massey (1991) highlights the problem of recurrently trying to draw boundaries to the conception of place and place-related concepts that, inherently, distinguishes between an inside (e.g., us) and an outside (e.g., them). She also supports that there is no need to conceptualize boundaries in order to define place, advocating that place is a process of social interactions. However, she asserts that those boundaries may be necessary for certain studies. It is within this reasoning that our study falls in: we attempt to spatially contextualize SoP, SC, and CE, to analyze the importance of their spatial relationship and their association. Thus, we do not deny the social dynamism of the studied concepts, but we need to spatially define individuals' spatial dimensions about significant places (i.e., SoP), meaningful social relationships (i.e., SC) and their spaces of engagement (i.e., CE) in a given time to evaluate their relationship.

The studies attempting to connect CE with environmental psychology (e.g., SoP) and/or social concepts (e.g., SC) have underestimated the geographical perspective that these concepts own, i.e., the spatial imprint that they acquire in the city context. Most of the studies that measure SoP (or related places concepts, e.g., place attachment (PA)) and SC are using pre-established administrative boundaries (i.e., neighborhood, parish, city, region, country) or individual-vague boundaries (i.e., home) as continuous and homogeneous containers (Mesch and Manor, 1998; Hidalgo and Hernández, 2001; Westlund et al., 2010). However, the citizens' perception of pre-established administrative boundaries can differ from the “real” one (Coulton et al., 2001; Montello et al., 2003) and, consequently, whole administrative boundaries might not cover the SoP, SC, and CE of all its dwellers. Hence, although studies systematically demonstrate that the sense of community (SC's dimension in Perkins and Long (2002)) is significant, positive and moderately strong related to forms of participation (Talò and Mannarini, 2015, p. 1) and some forms of SC are predictors of SoP (Mesch and Manor, 1998; Raymond et al., 2010); the positive spatial dimension and relationship of the three concepts (SoP, SC, and CE) has been briefly studied in the literature. In part, it is because of the gap between applications and methodologies to spatialize social concepts (Stedman, 2003). When we refer to spatializing a concept, we mean to transfer the non-spatial knowledge on SoP and SC to the geographical domain through GISc techniques.

The studied concepts (SoP, SC and CE) can be related to a human subjective meaning to a geographic area. Among the three concepts discussed in this study, SoP is the one in which the spatial dimension has been more thoroughly studied since its affective bonds are toward an area (Altman and Low, 1992). In turn, recent studies found out that a strong SoP helps to recall and describe memorable places (McCunn and Gifford, 2018), influencing the position and perspective that people infer in these visualizations (Newell and Canessa, 2018). The spatial dimension of social capital has also been analyzed (Rutten et al., 2010; Westlund et al., 2010; Foster et al., 2015), advocating for the potential of understanding and conceptualizing SC geographically (Putnam, 2000; Holt, 2008). However, some authors consider that geographical SC is ‘almost dead’ (see Radcliffe (2004)). Finally, CE and participation are inherently spatial (Pain and Kindon, 2007) and, consequently, influenced by social relations, time and space. The spatial dimension of CE (e.g., planning decisions or decision-making processes about communal spaces) has been established in administrative boundaries because of the availability of census and socioeconomic data in those areas (Dietz, 2002). However, this approach has probably hidden the spatial nature of CE associated with space, place and locality - essential characteristics to determine who is interested in the participatory processes and why (Carver, 2001). SoP and SC are strongly related in the non-spatial approach, as well as in the spatial one (Jorgensen, 2010; Jorgensen and Stedman, 2011; Acedo et al., 2017b), and the combination of both in a geographical area may well be the most meaningful places for a citizen (Lewicka, 2011). On the other hand, CE occurs within a particular spatial environment where an individual has informal cooperation ties and strong horizontal linkages, that is, SC (Zlatareva, 2008). Therefore, the research of the spatial dimension of the studied concepts (i.e., SoP, SC, and CE) could better explain their relationship (see Table 2) spatially (Table 3).

**Table 3:** Research hypotheses regarding the spatial relationship between sense of place, social capital and civic engagement.

Hypotheses number	Spatial hypotheses
H*s*_1_	A non-disjoint spatial relationship between SoP and SC spatial dimensions increases the influence of SoP on SC.
H*s*_2_	A non-disjoint spatial relationship between SC and CE spatial dimensions increases the influence of SC on CE.

## 3 Methodology

This methodology studies the effect of individuals' spatialities (i.e., SoP and SC) on CE behavior when a spatial relationship occurs between them. Thus, we establish a twofold methodology; firstly, to gather the spatial dimension of the three concepts mentioned above and, secondly, to evaluate their association through a geographical perspective using SEM.

### 3.1 Experimental design

In spite of all the critical implications that are related to mapping through GIS methodologies (see Elwood (2006)) and the inherent digital divide that these kinds of methodologies represent (Cruz-Jesus et al., 2012), we use a web map-based survey to gather all the (spatial) data of complex notions (SoP, SC, and CE). Thus, studied concepts derived from environmental, social, and participatory fields are artificially forced into geographic primitives (e.g., discrete points and/or polygons). Regarding this issue, Brown and Pullar (2012) compared studies with the two types of features and recommended the use of points instead of polygons in participatory GIS applications. Conversely, our approach uses polygons due to (1) the ease of implementation of “standard” drawing tools to define polygons and users' familiarity with that type of approach respect fuzzy designs (Huck et al., 2014); (2) the better encompass of a high range of spatial scales, (from an armchair to the whole earth (Tuan, 1978, p. 149)) and; (3) the better performance of polygon features when there is a limited spatial dataset (Brown and Pullar, 2012). Moreover, in the most recent and similar research to ours, Brown et al. (2015) use a Public Participation Geographic Information System (PPGIS) application to measure and map place attachment. They also define place attachment with polygon features from the minimum convex polygon of (at least) three points. However, the representation of geographically vague concepts (i.e., SoP, SC, and CE) through geographic primitives answer the need to classify the spatial relationship between them as positive or negative (i.e., whether there is a non-disjoint topological relationship or not, respectively).

**Figure 2:**
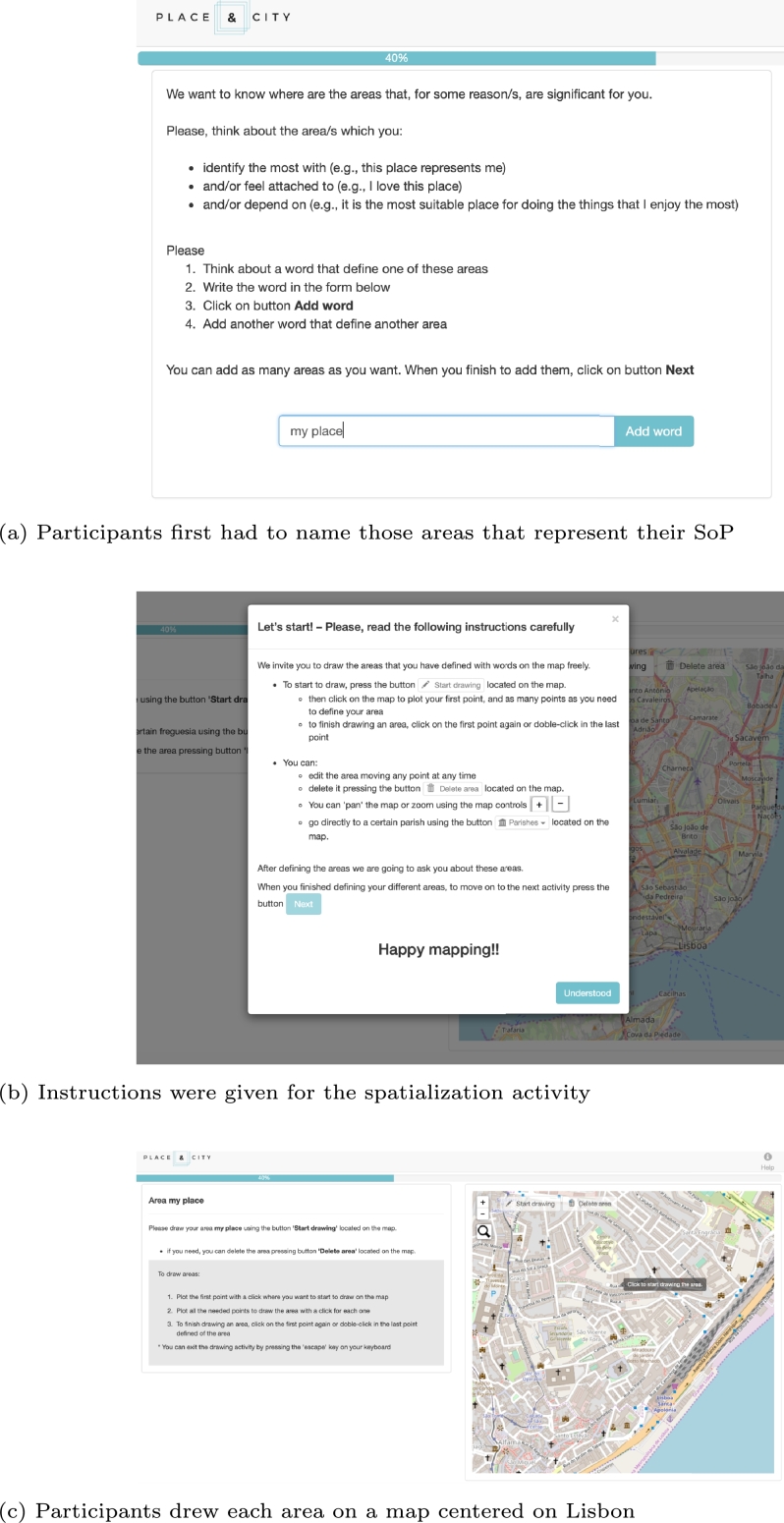

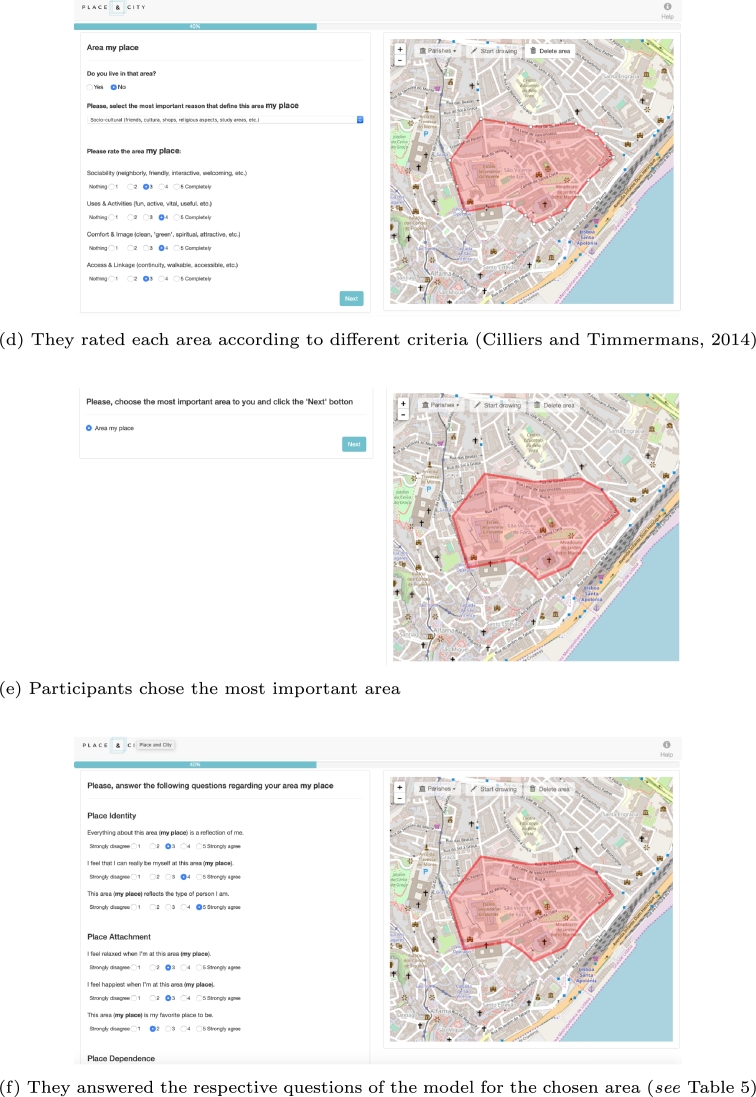
Web map-based survey flow to define SoP. [(d) Cilliers and Timmermans, 2014.]

The data were collected by applying a web map-based survey (Acedo et al., 2017a).^1^ All the data gathered are referenced to a singular geographical geometry along the city of Lisbon. The primary goal of this web map-based survey is to catch the spatial dimension of SoP, SC, and CE and measure their dimensions for a citizen in the city context. When we refer to the spatial dimension of a notion in this research, it is the geographical definition on a map of the area that covers the feelings, thoughts, and acts towards a place (i.e., SoP) a social group (i.e., SC) or engagement (i.e., CE). We introduced the three concepts (SoP, SC, and CE) and requested to the participants to think about their own places, social groups and spaces that comprise these three concepts, respectively. Fig. 2 shows the different steps that a participant faced when answering the web map-based survey, in this case, for the SoP concept. The survey had the same structure to define, spatialize and characterize the three concepts (i.e., SoP, SC, and CE).

**Figure 3:**
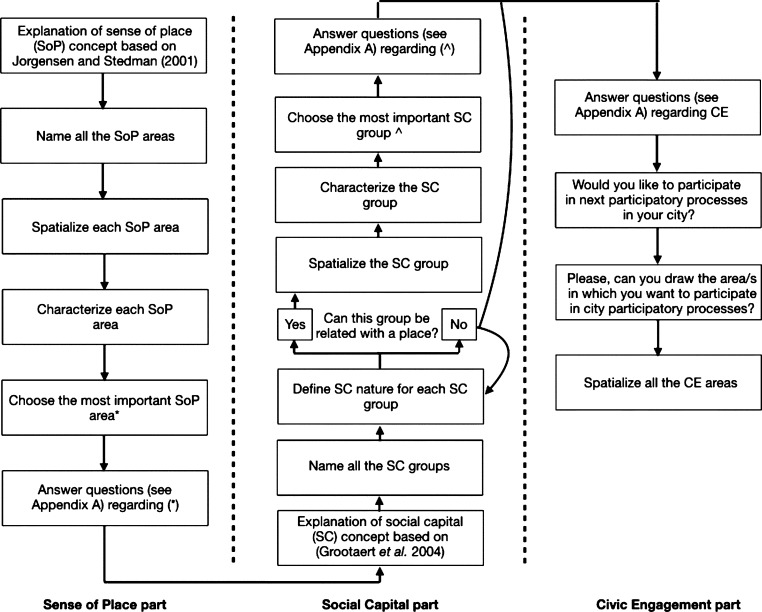
Schema of the application flow.

Each of the questions comprised in the tool were adapted from the literature (*see* Table 5). We tried to guide the respondents throughout the application precisely to improve the accuracy of the mapping activity (Brown and Pullar, 2012). At the end of the entire process, we gathered a spatial data (i.e., polygon) with qualitative information that attempts to ‘translate’ participants' rich socio-spatial understandings of SoP/SC and socio-spatial practices of CE. Some of them (i.e., those chosen as the most important by the participant) had qualitative information analyzed in an ordinal scale about the dimensions of SoP and SC. That ordinal information applies to measure the first-order dimensions of the model (see sub-section 5.1). We represented each variable through three questions; thus, SoP with three dimensions (i.e., PA, PI, and PD) needed nine questions, and SC (i.e., SoC, CEE, N, and CP) required twelve. All these questions are crucial to build the first-order dimensions that nourish the second-order reflective-formative constructs and, thus, the model. Fig. 3 shows (in-depth) all the sequence of steps that encompass the entire survey. Participants were also requested to contribute their sociodemographic information (age, gender, profession, income, and nationality). The municipality of Lisbon sent the survey to a database that contains a group of people engaged in the participatory processes in Lisbon; 373 people replied to the questionnaire in an approximately two-week period (i.e., 12 June to 2 July 2017 for this study). We did not use an ethical approval committee, instead, the municipality stated all the conditions and purposes of the collection process in the email that participants received. It was declared that all data collected would be treated with confidentiality and anonymity, and would not be used for commercial purposes or distributed to third parties.

## 4 Research model

**Figure 4:**
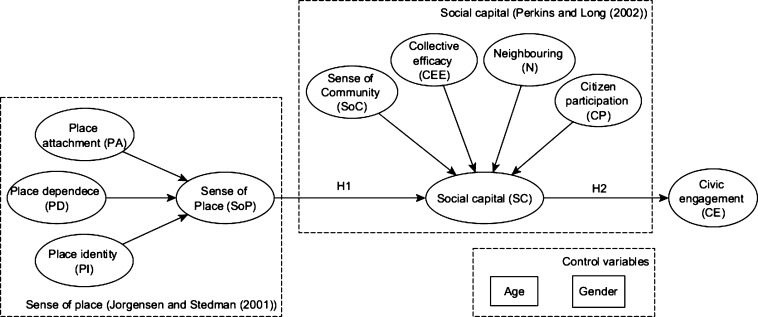
Research model.

This study integrates the SoP (Jorgensen and Stedman, 2001) and SC (Perkins and Long, 2002; Perkins et al., 2002) conceptualizations as predictors of CE (Son and Lin, 2008). SoP is integrated into the research model as a second-order reflective-formative construct determined by its three first-order dimensions: place attachment (PA), place identity (PI), and place dependence (PD). SC is another second-order reflective-formative construct determined by four first-order variables: sense of community (SoC), collective efficacy (CEE), neighboring (N), and citizen participation (CP). CE is the dependent construct of our model. Age and gender are included in the model as control variables on SC and CE. Fig. 4 shows the research model.

We use partial least squares structural equation modeling (PLS-SEM) (Hair et al., 2014) to evaluate the model since it is suitable for predictive analysis to test the hypotheses using empirical data (Hair et al., 2011). The measurement and structural model are estimated with SmartPLS 3.0 software (Ringle et al., 2015).

### 4.1 Introducing the spatial perspective in the research model

**Figure 5:**
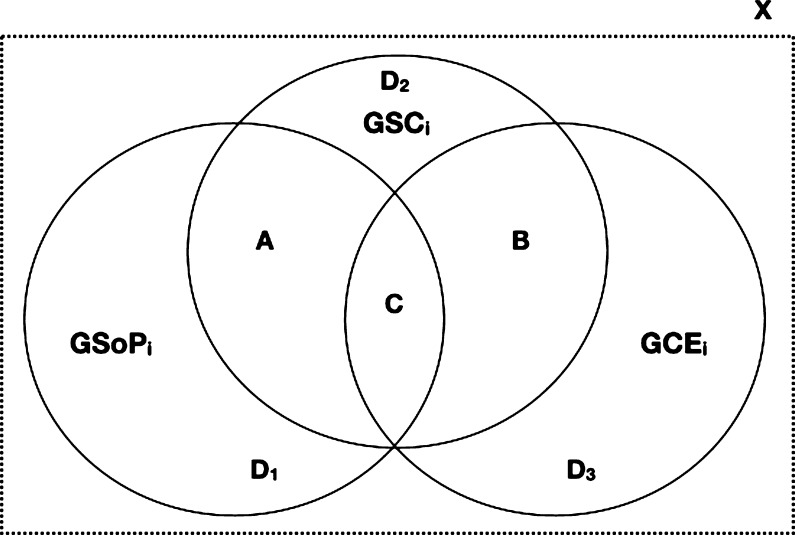
The spatial relationships between the three constructs: sense of place, social capital and civic engagement. Subset A represents the positive spatial relationship between GSoP and GSC, and B between GSC and GCE for a citizen. When both last statements occur for an individual is defined by the subset C. Finally, the last subset (D) is composed of those citizens without any positive spatial relationship between GSoP-GSC and GSC-GCE. Note: to make the document easier to read, we will treat GSo*P*_*i*_ as GSoP, GS*C*_*i*_ as GSC and GC*E*_*i*_ as GCE.

As mentioned above, SoP, SC, and CE exhibit spatial dimensions that can influence their mutual connections. Therefore, does the SoP, SC, and CE spatial relationship affect their association? Is there a spatial behavior between those concepts that can better explain their non-spatial association? This study analyzes the proposed research model (Fig. 4) for different subsets of respondents based on the diverse spatial relationship configurations that follow its constructors (SoP, SC and CE) for each citizen to answer these questions. This subsection wants to emphasize and operationalize the spatial dimension of the studied concepts (i.e., SoP, SC and CE) in order to study them in the research model (Fig. 4). The spatial characterization of the citizens' participants' subsets is based on the research of Egenhofer et al. (1994), which defined eight topological relationship types between two regions (polygons in this study) with connected boundaries (i.e., disjoint, meet, contains, covers, equal, overlap, inside and covered by). Seven of these spatial relationships follow a non-disjoint spatial behavior (coded as 1 for this study), that is assumed as the basis for classifying positive topological spatial relationships for SoP-SC, SC-CE and their own non-disjoint relationship. Fig. 5 summarizes both the different spatial relationships between the different constructs (SoP, SC and CE) and the resulting spatial subsets according to our model for each citizen ($c_{i}$) in the city context (*X*).

• A:
  (1) $$GSoP_{i}\cap GSC_{i}\neq \phi$$
• B:
  (2) $$GSC_{i}\cap GCE_{i}\neq \phi$$
• C:
  (3) $$GSoP_{i}\cap GSC_{i}\neq \phi \wedge GSC_{i}\cap GCE_{i}\neq \phi$$
• D:
  (4) $$GSoP_{i}\cap GSC_{i}=\phi \wedge GSC_{i}\cap GCE_{i}=\phi$$

where:

(5) $$GSOP_{i}=\overset{N}{\underset{j=1}{⋃}}GSOP_{ij}$$

(6) $$GSC_{i}=\overset{M}{\underset{k=1}{⋃}}GSC_{ik}$$

(7) $$GCE_{i}=\overset{O}{\underset{l=1}{⋃}}GCE_{il}$$

$c_{i}$ is a citizen.

i is an integer number between 1 and n, and n is the total number of citizens of a given city.

N, M, and O are positive integers, representing the total number of SoP, SC, and CE areas, respectively, for a citizen $c_{i}$.

GSo$P_{i}$ is the union of all individual Geographical Sense of Place(s) (GSo$P_{ij}$) for a citizen $c_{i}$.

GS$C_{i}$ is the union of all individual Geographical Social Capital(s) (GS$C_{ik}$) for a citizen $c_{i}$.

GC$E_{i}$ is the union of all individual Geographical Civic Engagement(s) (GC$E_{il}$) for a citizen $c_{i}$.

*X* is the surface of a given city.

We run the PLS-SEM using the four different datasets (i.e., A, B, C, and D) based on the disjoint and non-disjoint spatial relation of the citizens' geometries regarding SoP-SC and SC-CE (see Fig. 5).

## 5 Results

### 5.1 Data collection and measurement model

We received 373 complete answers. From these, 119 participants defined (at least) one area of each of SoP, SC, and CE. Participants were required to spatialize SoP areas because of its geographical nature, but SoP and CE can occur without being geographically defined (e.g., online social relationships). Therefore, a total of 119 citizens defined both SC and CE spatially, representing the working sample to conduct this study. Table 4 shows their demographics.

**Table 4:** Demographics of the sample for this study.

Demographic characteristics (N = 119)		Respondents	%
Age (years)	Less than 35	32	38.08
	Between 35 and 50	51	60.69
	More than 50	36	42.84
		
Gender	Female	63	74.97
	Male	56	66.64
		
Household monthly income (euros)	Less than 1000	23	27.37
	1000 - 1499	17	20.23
	1500 - 1999	10	11.9
	2000 - 2999	30	35.7
	3000 - 4999	10	11.9
	More than 5000	12	14.28
	N/A	17	20.23
		
Profession	Employed worker	72	85.68
	Freelance	17	20.23
	Retired	11	13.09
	Student	11	13.09
	Other	4	4.76
	Unemployed	4	4.76

The measurement model is evaluated using the full sample size (N = 119). SoP and SC are second-order reflective-formative constructs. CE is a first-order construct and the dependent variable in the model. We assess the measurement model following the approach of Hair et al. (2014) to evaluate that our measurement model is reliable. Table 5 shows all the questions from the literature used in the model and the Loadings associated (all above 0.7).

**Table 5:** Questions from the web map-based application and Loadings.

Construct	Question	Loading	Adapted from
Sense of place (SoP)	Place attachment		(Jorgensen and Stedman, 2001)
	I feel relaxed when I'm at this area (Y)	0.90	
	I feel happiest when I'm at this area (Y)	0.93	
	This area (Y) is my favorite place to be	0.87	
	Place dependence		
	This area (Y) is the best place for doing the things that I enjoy most	0.93	
	For doing the things that I enjoy most, no other place can compare to this area (Y).	0.82	
	This area (Y) is a good place to do the things I most like to do	0.89	
	Place identity		
	Everything about this area (Y) is a reflection of me	0.82	
	I feel that I can really be myself at this area (Y)	0.91	
	This area (Y) reflects the type of person I am	0.92	

Social capital (SC)	Sense of community		
	I feel like a member of the group Y	0.95	(Peterson et al., 2008)
	I belong to the group Y	0.96	
	I feel connected to the group Y	0.93	
	Collective efficacy/Empowerment		
	I think that a collective action from this group (Y) will increase chances of the local government changing their plans	0.91	(van Zomeren et al., 2008)
	I think that together (group (Y) members) we can change an issue	0.91	
	I think that it is important to get people in the group (Y) to help each other more	0.78	(Perkins and Long, 2002)
	Citizen participation		
	Have you attended a group (Y) meeting in the last 12 months?	0.91	(Ingrams, 2015)
	How often do you participate in the activities of the group (Y) in the last 12 months?	0.88	(Grootaert et al., 2004)
	To what extent did you participate in group (Y) decision-making in the last 12 months?	0.89	
	Neighbouring		
	Help a group (Y) member in an emergency	0.88	(Perkins and Long, 2002)
	Offer an advice on a personal problem of a group (Y) member	0.91	
	Discuss a problem with a group (Y) member	0.92	

Civic engagement (CE)	In the last 12 months, have you joined together with other people to address a community, local authority or governmental organization problems?	0.93	(Grootaert et al., 2004)
	In the last 12 months, have you talked with a community, local authority, or governmental organization about common problems?	0.93	
	In the last 12 months, have you worked with a community, local authority, or governmental, organization about common problems?	0.93	

Table 6 presents the quality assessment of the measurement model. For formative constructs, SoP and SC, we assess multicollinearity (Table 7). Both tables show the goodness of fit of our model.

**Table 6:** Quality assessment (square root of AVE in bold).

Constructs	CA	CR	AVE	1	2	3	4	5	6	7	8
1. Place attachment	0.88	0.93	0.81	**0.90**							
2. Place dependence	0.86	0.91	0.78	0.73	**0.88**						
3. Place identity	0.86	0.91	0.78	0.78	0.66	**0.88**					
4. Sense of community	0.94	0.96	0.90	0.34	0.32	0.32	**0.95**				
5. Collective efficacy	0.84	0.90	0.76	0.26	0.17	0.18	0.26	**0.87**			
6. Neighboring	0.89	0.93	0.82	0.27	0.20	0.20	0.48	0.36	**0.90**		
7. Citizen participation	0.88	0.92	0.80	0.12	0.04	0.13	0.35	0.27	0.46	**0.90**	
8. Civic engagement	0.92	0.95	0.86	-0.08	-0.19	-0.11	0.01	0.39	0.11	0.21	**0.93**

Notes: CA = Cronbach's Alpha, CR = Composite Reliability, AVE = Average Variance Extracted.

**Table 7:** Higher-order formative constructs. Inner VIF values (N=119).

Second-order formative constructs	First-order reflective constructs	VIF	Weights
Social capital (SC)	Sense of community	1.460	0.367^⁎⁎⁎^
	Collective efficacy	1.200	0.292^⁎⁎⁎^
	Neighboring	1.589	0.377^⁎⁎⁎^
	Citizen participation	1.332	0.336^⁎⁎⁎^
Sense of place (SoP)	Place attachment	3.177	0.398^⁎⁎⁎^
	Place dependence	2.210	0.348^⁎⁎⁎^
	Place identity	2.646	0.361^⁎⁎⁎^

### 5.2 Structural model

**Figure 6:**
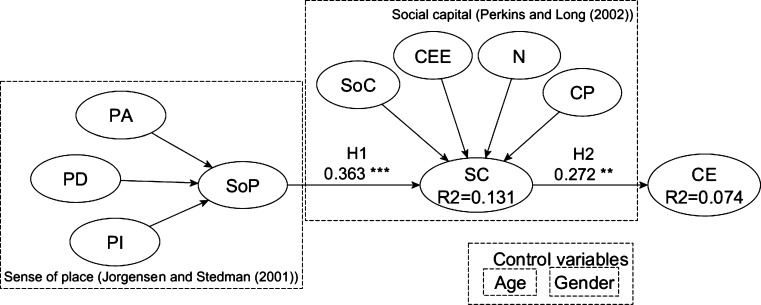
Structural model results.

The structural model is evaluated for the coefficient of determination ($R^{2}$) and the path coefficients (*β*). $R^{2}$ is a measure of the model's predictive power. Both SC and CE obtained $R^{2}$ values below the threshold of 0.25 (Fig. 6), which is described as weak predictive power (Henseler et al., 2009; Hair et al., 2014). The model path coefficients (*β*), its sign, and the statistical significance were assessed using the bootstrapping technique (Hair et al., 2014) with 5000 iterations. Age and gender were found to be not statistically significant on SC and CE.

Results of the structural model evaluation with the full sample size (N = 119) provide evidence to support the model (see Fig. 6). The results reveal that the three SoP variables (i.e., place attachment (PA), dependence (PD) and identity (PI)) significantly explain the construct. Hence, this study validates the conceptualization of SoP by Jorgensen and Stedman (2001) as was performed by Pretty et al. (2003). The calculated model also provides evidence that the four first-order variables (i.e., sense of community (SoC), collective efficacy (CEE) neighboring (N) and citizen participation (CP)) significantly explain SC, supporting Perkins and his colleagues' (Perkins et al., 2002; Perkins and Long, 2002) conceptualization of SC. Finally, the results from the structural model (Fig. 6) disclose that SoP has a positive effect on SC ($H_{1}$) and, in turn, SC has a positive effect on CE ($H_{2}$). The next subsection will analyze the acceptance of hypotheses $H_{s1}$ and $H_{s2}$ based on $H_{1}$ and $H_{2}$, respectively, for the subsets derived from the spatial relationship between SoP, SC, and CE.

### 5.3 A geographical evaluation of the structural model

As mentioned in previous discussions, one of the main goals of this study is the inclusion and analysis of the spatial relationship between GSoP, GSC and GCE in our model to prove the importance of the spatial dimension of studied concepts in urban processes and dynamics. Based on the data gathered and methodology followed we obtained the following spatial subsets:

• A:
  (8) $$GSoP_{i}\cap GSC_{i}\neq \phi (N=57)$$
• B:
  (9) $$GSC_{i}\cap GCE_{i}\neq \phi (N=76)$$
• C:
  (10) $$GSoP_{i}\cap GSC_{i}\neq \phi \wedge GSC_{i}\cap GCE_{i}\neq \phi (N=44)$$
• the disjoint one D:
  (11) $$GSoP_{i}\cap GSC_{i}=\phi \wedge GSC_{i}\cap GCE_{i}=\phi (N=34)$$

**Figure 7:**
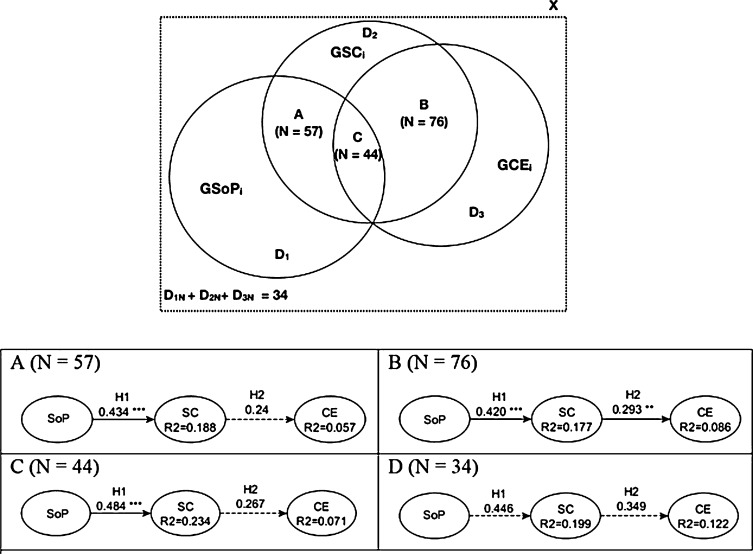
The spatial relationships between the three constructs according to our model and their respective results in the structural model. Discontinue lines mean statistically not significant.

Fig. 7 illustrates the schema of the resulting datasets derived from our model (Fig. 4) and the different structural model results for the non-disjoint and disjoint subsets (A, B, C and D). Table 8 and Table 9 provide *β* and $R^{2}$ results for the four spatial datasets, respectively.

**Table 8:** Structural model evaluation (*β* values) for the sample and related subsets.

Path	All obs. (119)	Spatial related subsets (# observations)			
		A - (57)	B - (76)	C - (44)	D - (34)
SOP → SC (*H*_1_)	0.363^⁎⁎⁎^	0.434^⁎⁎⁎^	0.42^⁎⁎⁎^	0.484^⁎⁎⁎^	0.446
SC → CE (*H*_2_)	0.272^⁎⁎^	0.24	0.293^⁎⁎^	0.267	0.349
Indirect effect (SOP → CE)	0.099^⁎^	0.104	0.123^⁎⁎^	0.129	0.156

Notes: Significant at ^⁎^10%; ^⁎⁎^5%; ^⁎⁎⁎^1%.

**Table 9:** *R*^2^ square values for the sample and related subsets.

Dataset	*R*^2^ (SC)	*R*^2^ (CE)
All obs. (119)	0.131	0.074
A (57)	**0.188**	0.057
B (76)	0.177	**0.086**
C (44)	0.234	0.071
D (34)	0.199	0.122

Hypothesis $H_{1}$ is fully supported for all three datasets where a non-disjoint relationship exists (i.e., A, B and C) but is not statistically significant for the disjoint subset (i.e., D) (see Table 8). $H_{2}$ is just supported for one of the three datasets with non-disjoint spatial behavior (i.e., B). For datasets A and C, $H_{2}$ is not statistically significant, therefore not supported. In the cases where $H_{1}$ and $H_{2}$ are statistically significant, the influence of SoP on SC and SC on CE is stronger than the dataset with all the observations (see Table 8). The indirect effect of SoP on CE is also statistically significant for one of the three geographical related datasets (i.e., B). Subset D represents the citizens who defined their geometries regarding SoP-SC and SC-CE as being disjoint. For this group (i.e., D), none of the path coefficients were statistically significant. Table 10 shows a summary of the supported and rejected hypotheses of this study.

**Table 10:** Summary of supported (y) and rejected (n) hypothesis.

Hypothesis	All observations	Non-disjoint subsets			Disjoint subset
		A	B	C	D
*H*_1_	y	y	y	y	n
*H*_2_	y	n	y	n	n

Therefore, the better results for the $H_{1}$ and $H_{2}$ path coefficients values (Table 8) and associated $R^{2}$ (Table 9) in relation to subsets A (GSOP and GSC non-disjoint relationship) and B (GSC and GCE non-disjoint relationship), respectively (see bold results in Table 9), allow us to support $H_{s1}$ and $H_{s2}$. This finding provides evidence that the geographical component plays a critical role in the statistical significance of the path coefficients in the prediction of CE, i.e., the influence on SoP to SC and SC on CE are statistically better explained when there is a non-disjoint spatial relationship between them.

## 6 Discussion

This research attempts to validate the importance of SoP and SC spatial relationships to explain CE at the individual level. These spatial interactions define new approaches to better understanding the city's social realm from the geographic study of social concepts. We highlight the suitability of these social concepts to encapsulate human notions that can be rendered on a map and we elucidate connections with the previous understanding of cities as place networks (Massey, 1994; Roche, 2016; Acedo et al., 2018). Bridging (spatial) scholarship within social theory and environmental psychology through a participatory methodology using GISc techniques in a continually shifting city network environment (Murdoch, 1998; Latour, 2005; Duff, 2011), expands the participatory research agenda and embraces two general areas (i.e., GISc and humanities) that, unfortunately, has been rarely analyzed together in depth (Bodenhamer et al., 2010). Surely, this carelessness has been mainly due to the dynamism and the vague nature of those rich socio-spatial concepts (i.e., SoP, SC and CE) and the considerable difficulty of GISc techniques to embed their fuzzy perseverance (Coulton et al., 2001; Huck et al., 2014).

The results of our model shows low $R^{2}$ values for both SC and CE in all models (Table 9) are in line with other studies that have reported similar $R^{2}$ values; for instance, in the study of civic activity (Lewicka, 2005) and pro-environmental CE (Buta et al., 2014), the $R^{2}$ values found were smaller than 0.16 and less than 0.33, respectively. Thus, this study introduces the spatial component as part of the analysis to try to overcome this issue and to obtain better explanatory models. Our findings show that when there is a non-disjoint spatial relationship between the studied concepts (SoP, SC, and CE), the corresponding model performs a better statistical description of their associations.

SoP and SC display the most consistent relationship of the model. This relationship is statistically significant for all the subsets except D. Furthermore, SC is better explained by SoP when there is a non-disjoint relationship between both concepts' spatial dimensions (i.e., GSoP and GSC). The results of this study are in line with the conceptualizations of Acedo et al. (2017b) advocating for the strong spatial relationship of these two concepts and Jorgensen (2010), who assures the mutual spatial behavior between the two concepts. Independently of their spatial nature, it is clear from the findings of our study that the non-disjoint relationship between SoP and SC strengthens the explanation of SC by SoP. Surprisingly, the other positive geographical related spatial subsets (i.e., B and C) also show significant and better values than the entire dataset, showing that $H_{1}$ performs better when a positive (non-disjoint) spatial interaction occurs in the model. Only for subset D (disjoint subset) is the relationship from SoP to SC not statistically significant. Regarding the method to statistically evaluate the different subsets (i.e., PLS-SEM), it is worth noting that, to the best of our knowledge, this is the first study to add the spatial relationship between constructs into a model. Jorgensen and Stedman (2011) integrate the spatial and physical features of places with attitude and behavioral variables in models of this type, but the specific study of the spatial dimension of model' constructs has never been investigated to date.

Overall, the relationship between SC and CE is not as strong as that between SoP and SC. Interestingly, the only geographical subset that has statistical significance is B (i.e., when there is a positive spatial relationship between SC and CE for a citizen). Thus, to explain how SC influences CE, it is interesting to highlight that its association is stronger when there is a non-disjoint relationship between their geographical areas. This finding is in consonance, in part, with studies assuring that participation is likely to occur in small-group situations (Rydin and Pennington, 2011), where the citizen has a higher identification and satisfaction with the group (Bernardo and Palma-Oliveira, 2016). Accordingly, this research contributes by highlighting the importance of these group's relationships (SC) being located in the same place where for instance, the participatory or planning process is taking place to have better CE performance. The other two subsets (i.e., A and C) and subset D do not show statistical significance in the relationship between SC and CE.

We assume that there is a difficulty to switch current participatory geographies (i.e., the spaces where the governments are setting up participatory processes) based on administrative boundaries to one based on common citizens' spatialities. The underlying reason to use those administrative boundaries is to find out the percentage of the participatory results upon census and socioeconomic data in those specific areas. However, the understanding of the spatial relationship between SoP, SC and CE establishes novel spatial scenes based on human-city interactions. There is a potential for understanding the spatial relationships of social concepts (i.e., SoP, SC and CE) to provide alternate or completely new units of analysis for citizen participation. The definition and mapping of these individuals' spatialities allow a more citizen-focused representation of the city.

Given the importance of CE to the process of city governance (Adler and Goggin, 2005) and the challenge to quantify the relationship between a physical environment and a person's emotional experience (Hu and Chen, 2018), the three conceptualizations, at the individual level, draw a potential for adopting new methodologies to represent the urban organization of cities. For example, a government could adopt a new perspective to interacting with citizens based on citizens' relationship toward a certain geographical area (i.e., SoP), their significant social relationships (i.e., SC) or simply the areas within an individual wanting to participate (i.e., CE). Moreover, the spatial relationship of the three concepts could possibly provide a framework for the municipal organization of service provision. The addition of these spatial definitions of SoP, SC, and CE could revitalize the current top-down jurisdictional approach in formal participation processes to recruit input from broader, more diverse citizen groups, nourishing more holistic master plans.

This study is focused on the urban context and their results are constrained on the circumstances of the city of Lisbon. Although the results cannot be directly generalized for other cities, the methodology and the open source tools used in this study allow their replicability in other urban contexts. In turn, the sample size characterized by the area of study (N = 119) and the derived smaller subsets based on the constructs' geographical behavior could also represent a limitation to conduct the study. Ideally, larger sample sizes lead to more accurate results. Other approaches to gather SoP (Jenkins et al., 2016) and SC (Antoci et al., 2015) data through social network analysis are appearing in the last years. Unlike our approach, perhaps, these techniques can provide a quick approach to the concept as well as to gather a massive related dataset. However, it remains unclear how these techniques can infer the specific spatial area (polygon) for citizens' SoP or to measure the dimensions of SC from social network analysis to relate both pieces of information for a single citizen. Conversely, our approach goes straight to the point with the spatial representation and measurement of SoP, SC, and CE at the individual level.

Some non-representational theorists have defended the necessity of not emphasizing representation as the primary step to extract knowledge (Dewsbury, 2003; Thrift, 2008), especially in social theory, attending to the constantly relational nature of actors' interaction. We do not deny this nature, but our study needs of a “spatial picture” of the individuals' spatialities in a given time (e.g., 12 June to 2 July 2017 for this study) in order to evaluate their spatial relationship in socio-spatial processes such as participatory processes. In turn, the authors of this study acknowledge the dynamism, time-dependent, and scale variability of studied concepts (i.e., SoP, SC and CE) as a limitation of this study, highlighting the need for longitudinal time-series studies and a dynamic collection of social data for a better comprehension of the phenomena. In this respect, mapping activity using polygons can also exhibit either spatial and/or scalar ambiguity (Huck et al., 2014). Moreover, we already argued about the relative accuracy in defining the spatial dimension through polygons for concepts such as SoP, SC, and CE. Thereby, our approach can be understood as an attempt to study the spatial dimension of those concepts and their spatial relationships. However, based on the results of this paper, the mapping activity through polygons attains better goodness of fit in the model (Fig. 4) when there is a positive spatial relationship. Therefore, our approach to mapping the spatial dimension of those concepts (i.e., SoP, SC and CE) substantially cover their spatial association and trace a possible valid path to operationalize their spatial imprint, and possibly other social concepts, in the city context.

## 7 Conclusions

This research connects citizens' areas of significant interactions (i.e., SC), positive environmental attitude towards places (i.e., SoP) and engagement to participate in community, society, planning and governmental issues (i.e., CE). The spatial data gathered from the web map-based application allows us to attempt the spatialization of citizens' SoP, SC, and CE, psychological, social and participatory concepts that are critical in citizens' daily tasks and interactions. The findings of this study demonstrate spatiality of and spatial relationships among SoP, SC, and CE, based on a GIS-based analysis of data collected through a participatory methodology. The knowledge and management of these interactions, and where their spatial relationships occur, creates an occasion that provides fruitful social-spatial data for other areas of knowledge such as planning or participation. To some extent, we are setting up the foundations of new *geographies of engagement* for all the stakeholders of a city. Furthermore, the rainbow of applications that may profit from such an understanding of space is wide, extending from location-based services to community detection and even citizen science processes (Haywood, 2014; Newman et al., 2016). This article highlights the role of the geographical perspective in taking another step forward to better understand citizens' social synergies in the urban context. Specifically, how GIS techniques can be used to attempt the operationalization of rich-complex human based concepts such as SoP, SC, and CE. On the other hand, the use of PLS-SEM to explore the impact of spatial components in combination with non-spatial variables has been rarely used in the literature (Jorgensen and Stedman, 2011). The method used in this research discloses the potential of introducing spatial perspectives in PLS-SEM models. Future work can be along the lines of adding the relevant features enclosed in the spatial dimension of studied concepts into the research model to investigate how and what physical space is valued and influences the studied concepts (i.e., SoC, SC, and CE).

### 7.1 Notes to advance in the spatial acquisition of social concepts

We foresee a significant potential to truly appreciate the spatial dimension of social concepts as spatial (forgive the repetition), i.e., to take a step further, recognizing and operationalizing the crucial matter of the spatial domain in social theory. This is not just to discuss or embed results in administrative boundaries, but to really assign the spatial dimension of social concepts in the studies' methodology section. Unfortunately, this research is one of the few studies of the long way to go in the meaningful operationalization of the social concepts spatial dimension in the urban context. Once this process is normalized and dynamically updated, we will be able to disclose the suitability of including the geographical perspective in, for instance, social, planning and participatory studies. There is a shortage of empirical research on the interactions between people and places. Therefore, this study calls for efforts that bridge multiple academic communities to open innovative avenues for understanding social-spatial behaviors, the outcomes of such encounters, and their addition in city' procedures such as participatory processes. The spatial understanding of that synergy highlights a promising area of future scholarship.

## Declarations

### Author contribution statement

Albert Acedo: Conceived and designed the experiments; Performed the experiments; Analyzed and interpreted the data; Contributed reagents, materials, analysis tools or data; Wrote the paper.

Tiago Oliveira: Contributed reagents, materials, analysis tools or data.

Mijail Naranjo-Zolotov: Performed the experiments; Analyzed and interpreted the data; Wrote the paper.

Marco Painho: Conceived and designed the experiments.

### Funding statement

Albert Acedo Sánchez and Mijail Naranjo-Zolotov gratefully acknowledge funding from the European Commission through the GEO-C project (H2020-MSCA-ITN-2014, Grant Agreement Number 642332, http://www.geo-c.eu/).

### Competing interest statement

The authors declare no conflict of interest.

### Additional information

No additional information is available for this paper.

## Footnotes

1 https://placeandcity.com [accessed on 25th of August 2018].

## References

[br0010] Acedo A., Mendoza G., Painho M., Casteleyn S., Bregt A., Sarjakoski T., Lammeren R., Rip F. (2017). One tool to spatialize all: sense of place, social capital and civic engagement. Societal Geo-Innovation: Short Papers, Posters and Poster Abstracts of the 20th AGILE Conference on Geographic Information Science.

[br0020] Acedo A., Painho M., Casteleyn S. (2017). Place and city: operationalizing sense of place and social capital in the urban context. Trans. GIS.

[br0030] Acedo A., Painho M., Casteleyn S., Roche S. (2018). Place and city: toward urban intelligence. ISPRS Int. J. Geo-Inf..

[br0040] Adler R.P., Goggin J. (2005). What do we mean by “civic engagement”?. J. Transformat. Educ..

[br0050] Agnew J.A. (2002). Place and Politics in Modern Italy.

[br0060] Agnew J.A., Agnew John, Livingstone David N. (2011). Space and place. Handbook of Geographical Knowledge.

[br0070] Ajzen I., Fishbein M. (1975). Attitude-behavior relations: a theoretical analysis and review of empirical research. Psychol. Bull..

[br0080] Altman I., Low S.M. (1992). Place Attachment.

[br0090] Antoci A., Sabatini F., Sodini M. (2015). Online and offline social participation and social poverty traps: can social networks save human relations?. J. Math. Sociol..

[br0100] Anton C.E., Hons B.A. (2016). Home Sweet Home: an Examination of the Relationship Between Place Attachment and Place-Protective Actions.

[br0110] Aricat R.G., Ling R., Wei R. (2016). Civic engagement in Myanmar: the promise and threat of mobile communication and the Internet. Mobile Communication in Asia: Local Insights, Global Implications.

[br0120] Bernardo F., Palma-Oliveira J.M. (2016). Identification with the neighborhood: discrimination and neighborhood size. Self and Identity.

[br0130] Bodenhamer D.J., Corrigan J., Harris T.M. (2010). The Spatial Humanities.

[br0160] Brown G.G., Pullar D.V. (2012). An evaluation of the use of points versus polygons in public participation geographic information systems using quasi-experimental design and Monte Carlo simulation. Int. J. Geogr. Inf. Sci..

[br0140] Brown G., Raymond C.M., Corcoran J. (2015). Mapping and measuring place attachment. Appl. Geogr..

[br0150] Brown G., Reed P., Harris C. (2002). Testing a place-based theory for environmental evaluation: an Alaska case study. Appl. Geogr..

[br0170] Buta N., Holland S.M., Kaplanidou K. (2014). Local communities and protected areas: the mediating role of place attachment for pro-environmental civic engagement. J. Outdoor Recreat. Tour..

[br0180] Cadman L. (2009). Nonrepresentational theory/nonrepresentational geographies. International Encyclopedia of Human Geography.

[br0190] Carver S. (2001). Participation and geographical information: a position paper. ESFNSF Workshop on Access to Geographic Information and Participatory Approaches Using Geographic Information.

[br0200] Cegarra-Navarro J.G., Garcia-Perez A., Moreno-Cegarra J.L. (2014). Technology knowledge and governance: empowering citizen engagement and participation. Gov. Inf. Q..

[br0210] Chan M. (2018). Digital communications and psychological well-being across the life span: examining the intervening roles of social capital and civic engagement. Telemat. Inform..

[br0220] Chen J. (2016). Can online social networks foster young adults' civic engagement?. Telemat. Inform..

[br0230] Cilliers E.J., Timmermans W. (2014). The importance of creative participatory planning in the public place-making process. Environ. Plan. B, Plan. Des..

[br0240] Coulton C.J., Korbin J., Chan T., Su M. (2001). Mapping residents' perceptions of neighborhood boundaries: a methodological note. Am. J. Community Psychol..

[br0250] Cruz-Jesus F., Oliveira T., Bacao F. (2012). Digital divide across the European Union. Inf. Manag..

[br0260] DeLyser D., Sui D. (2014). Crossing the qualitative-quantitative chasm III: enduring methods, open geography, participatory research, and the fourth paradigm. Prog. Hum. Geogr..

[br0270] Dewsbury J.D. (2003). Witnessing space: ‘Knowledge without contemplation’. Environ. Plan. A.

[br0280] Dietz R.D. (2002). The estimation of neighborhood effects in the social sciences: an interdisciplinary approach. Soc. Sci. Res..

[br0290] Duff C. (2011). Networks, resources and agencies: on the character and production of enabling places. Health Place.

[br0300] Egenhofer M.J., Clementini E., di Felice P. (1994). Research paper. Int. J. Geogr. Inf. Syst..

[br0310] Elwood S. (2006). Critical issues in participatory GIS: deconstructions, reconstructions, and new research directions. Trans. GIS.

[br0320] Fishbein M., Ajzen I. (1975). Belief, Attitude, Intention, and Behavior: An Introduction to Theory and Research.

[br0330] Foster K.A., Pitner R., Freedman D.A., Bell B.A., Shaw T.C. (2015). Spatial dimensions of social capital. City Community.

[br0340] Grootaert C., Narayan D., Jones V.N., Woolcock M. (2004). Measuring Social Capital: An Integrated Questionnaire.

[br0350] Hair J., Hult T., Ringle C., Sarstedt M. (2014). A Primer on Partial Least Squares Structural Equation Modeling (PLS-SEM).

[br0360] Hair J.F., Ringle C.M., Sarstedt M. (2011). PLS-SEM: indeed a silver bullet. J. Mark. Theory Pract..

[br0370] Haywood B.K. (2014). A “sense of place” in public participation in scientific research. Sci. Educ..

[br0380] Henseler J., Ringle C.M., Sinkovics R.R. (2009). The use of partial least squares path modeling in international marketing. Adv. Int. Mark..

[br0390] Hidalgo M.C., Hernández B. (2001). Place attachment: conceptual and empirical questions. J. Environ. Psychol..

[br0400] Holt L. (2008). Embodied social capital and geographic perspectives: performing the habitus. Prog. Hum. Geogr..

[br0410] Hu M., Chen R. (2018). A framework for understanding sense of place in an urban design context. Urban Sci..

[br0420] Huck J.J., Whyatt J.D., Coulton P. (2014). Spraycan: a PPGIS for capturing imprecise notions of place. Appl. Geogr..

[br0430] Hunter B., Gullotta T.P., Walberg H.J., Weissberg R.P. (2016). Social capital: models and efforts to build and restore among marginalized individuals and communities. Social Capital and Community Well-Being.

[br0440] Ingrams A. (2015). Mobile phones, smartphones, and the transformation of civic behavior through mobile information and connectivity. Gov. Inf. Q..

[br0450] Jenkins A., Croitoru A., Crooks A.T., Stefanidis A. (2016). Crowdsourcing a collective sense of place. PLoS ONE.

[br0460] Jorgensen B.S. (2010). Subjective mapping methodologies for incorporating spatial variation in research on social capital and sense of place. Tijdschr. Econ. Soc. Geogr..

[br0470] Jorgensen B.S., Stedman R.C. (2001). Sense of place as an attitude: lakeshore owners attitudes toward their properties. J. Environ. Psychol..

[br0480] Jorgensen B.S., Stedman R.C. (2011). Measuring the spatial component of sense of place: a methodology for research on the spatial dynamics of psychological experiences of places. Environ. Plan. B, Plan. Des..

[br0490] Kahila M., Kyttä M., Geertman S., Stillwell J. (2009). SoftGIS as a bridge-builder in collaborative urban planning. Planning Support Systems Best Practice and New Methods.

[br0500] Kil N., Holland S., Stein T. (2014). Place meanings and participatory planning intentions. Soc. Nat. Resour..

[br0510] Kitchin R., Gleeson J., Dodge M. (2013). Unfolding mapping practices: a new epistemology for cartography. Trans. Inst. Br. Geogr..

[br0520] Kyttä M., Kahila M. (2011). SoftGIS methodology—building bridges in urban planning. GIM Int. (Glob. Mag. Geomat.).

[br0530] Latour B. (2005). Reassembling the Social.

[br0540] Law J. (2008). On sociology and STS. Sociol. Rev..

[br0550] Lewicka M. (2005). Ways to make people active: the role of place attachment, cultural capital, and neighborhood ties. J. Environ. Psychol..

[br0560] Lewicka M. (2011). Place attachment: how far have we come in the last 40 years?. J. Environ. Psychol..

[br0570] Lewicka M. (2013). Place inherited or place discovered? Agency and communion in people-place bonding. Estud. Psicol..

[br0580] Lin C.C., Lockwood M. (2014). Forms and sources of place attachment: evidence from two protected areas. Geoforum.

[br0590] Lussault M. (2007). L'Homme spatial. La construction sociale de l'espace humain: La construction sociale de l'espace humain.

[br0600] Mahmoudi Farahani L. (2016). The value of the sense of community and neighbouring. Hous. Theory Soc..

[br0610] Manzo L.C. (2005). For better or worse: exploring multiple dimensions of place meaning. J. Environ. Psychol..

[br0620] Manzo L.C., Perkins D.D. (2006). Finding common ground: the importance of place attachment to community participation and planning. J. Plan. Lit..

[br0630] Massey D. (1991). A global sense of place. Marx. Today.

[br0640] Massey D. (1994). Space, Place, and Gender.

[br0650] McCunn L.J., Gifford R. (2018). Spatial navigation and place imageability in sense of place. Cities.

[br0660] Mesch G.S., Manor O. (1998). Social ties, environmental perception, and local attachment. Environ. Behav..

[br0670] Mohammadi S.H., Norazizan S., Shahvandi A.R. (2011). Civic engagement, citizen participation and quality of governance in Iran. J. Hum. Ecol..

[br0680] Montello D.R., Goodchild M.F., Gottsegen J., Fohl P. (2003). Where's downtown?: behavioral methods for determining referents of vague spatial queries. Spat. Cogn. Comput..

[br0690] Moro G. (2010). Civic action. International Encyclopedia of Civil Society.

[br0700] Murdoch J. (1998). The spaces of actor-network theory. Geoforum.

[br0710] Naranjo Zolotov M., Oliveira T., Casteleyn S. (2018). E-participation adoption models research in the last 17 years: a weight and meta-analytical review. Comput. Hum. Behav..

[br0720] Naranjo-Zolotov M., Oliveira T., Cruz-Jesus F., Martins J., Gonçalves R., Branco F., Xavier N. (2019). Examining social capital and individual motivators to explain the adoption of online citizen participation. Future Gener. Comput. Syst..

[br0730] Newell R., Canessa R. (2018). From sense of place to visualization of place: examining people-place relationships for insight on developing geovisualizations. Heliyon.

[br0740] Newman G., Chandler M., Clyde M., McGreavy B., Haklay M., Ballard H., Gray S., Scarpino R., Hauptfeld R., Mellor D., Gallo J. (2016). Leveraging the power of place in citizen science for effective conservation decision making. Biol. Conserv..

[br0750] Pain R., Kindon S. (2007). Participatory geographies. Environ. Plan. A.

[br0760] Perkins D.D., Brown B.B., Taylor R.B. (1996). The ecology of empowerment: predicting participation in community organizations. J. Soc. Issues.

[br0770] Perkins D.D., Hughey J., Speer P.W. (2002). Community psychology perspectives on social capital theory and community development practice. J. Community Dev. Soc..

[br0780] Perkins D.D., Long D.A., Fischer A., Sonn C., Bishop B. (2002). Neighborhood sense of community and social capital: a multi-level analysis. Psychological Sense of Community: Research, Applications and Implications.

[br0790] Peterson N.A., Speer P.W., McMillan D.W. (2008). Validation of a brief sense of community scale: confirmation of the principal theory of sense of community. J. Community Psychol..

[br0800] Pile S. (2010). Emotions and effect in recent human geography. Trans. Inst. Br. Geogr..

[br0810] Pretty G.H., Chipuer H.M., Bramston P. (2003). Sense of place amongst adolescents and adults in two rural Australian towns: the discriminating features of place attachment, sense of community and place dependence in relation to place identity. J. Environ. Psychol..

[br0820] Proshansky H.M., Fabian A.K., Kaminoff R. (1983). Place-identity: physical world socialization of the self. J. Environ. Psychol..

[br0830] Putnam R.D. (2000). Bowling Alone: The Collapse and Revival of American Community, vol. 747.

[br0840] Radcliffe S.A. (2004). Geography of development: development, civil society and inequality – social capital is (almost) dead?. Prog. Hum. Geogr..

[br0850] Raymond C.M., Brown G., Weber D. (2010). The measurement of place attachment: personal, community, and environmental connections. J. Environ. Psychol..

[br0860] Ringle C.M., Wende S., Becker J.M. (2015). SmartPLS 3.

[br0870] Roche S. (2016). Geographic information science II: less space, more places in smart cities. Prog. Hum. Geogr..

[br0880] Rosenberg M.J. (1960). Cognitive, affective, and behavioral components of attitudes. Attitude Organization and Change.

[br0890] Rutten R., Westlund H., Boekema F. (2010). The spatial dimension of social capital. Eur. Plan. Stud..

[br0900] Rydin Y., Pennington M. (2011). Public participation and local environmental planning: the collective action problem and the potential of social capital. Local Environ.: Int. J. Justice Sustain..

[br0910] Sheedy A., Mackinnon P., Pitre S., Watling J. (2008). Handbook on Citizen Engagement: Beyond Consultation.

[br0920] Son J., Lin N. (2008). Social capital and civic action: a network-based approach. Soc. Sci. Res..

[br0930] Stedman R.C. (2003). Is it really just a social construction?: the contribution of the physical environment to sense of place. Soc. Nat. Resour..

[br0940] Sui D., DeLyser D. (2012). Crossing the qualitative-quantitative chasm I: hybrid geographies, the spatial turn, and volunteered geographic information (VGI). Prog. Hum. Geogr..

[br0950] Talò C., Mannarini T. (2015). Measuring participation: development and validation the participatory behaviors scale. Soc. Indic. Res..

[br0960] Thrift N. (2008). Non-representational Theory: Space, Politics, Affect.

[br0970] Trentelman C.K. (2009). Place attachment and community attachment: a primer grounded in the lived experience of a community sociologist. Soc. Nat. Resour..

[br0980] Tuan Y.F. (1978). Space and Place: The Perspective of Experience, vol. 7.

[br0990] UNDP Evaluation Office (2002). Civic Engagement, Essentials No. 8.

[br1000] Westlund H., Rutten R., Boekema F. (2010). Social capital, distance, borders and levels of space: conclusions and further issues. Eur. Plan. Stud..

[br1010] Zlatareva M. (2008). Promoting Civic Engagement in a Post- Totalitarian and EU Accession Context: a Case From Bulgaria.

[br1020] van Zomeren M., Postmes T., Spears R. (2008). Toward an integrative social identity model of collective action: a quantitative research synthesis of three socio-psychological perspectives. Psychol. Bull..

